# Development and validation of a nomogram for predicting poor operative visibility during FESS in Chinese adult patients with CRS

**DOI:** 10.3389/fmed.2024.1344661

**Published:** 2024-04-29

**Authors:** Deping Sun, Yalan Liang, Fuwei Yang, Lan Liu, Xuemei Mao, Xiaoli Xu

**Affiliations:** Department of Otorhinolaryngology Head and Neck Surgery, The Fourth Clinical College of Chongqing Medical University, Chongqing, China

**Keywords:** chronic rhinosinusitis, functional endoscopic sinus surgery, operative visibility, risk factors, nomogram

## Abstract

**Objective:**

The purpose of this study is to develop and evaluate a nomogram that is capable of predicting poor operative visibility during functional endoscopic sinus surgery.

**Method:**

To identify potential risk factors, patients with chronic rhinosinusitis who underwent functional endoscopic sinus surgery (FESS) between January 2019 and December 2022 were selected from our hospital’s electronic medical record system. Data on general patient information, clinical manifestations, clotting-related test indices, Lund-Machay score of sinuses CT scanning, Lund-kennedy score of nasal endoscopies, anesthesia methods, intraoperative blood pressure and heart rate, and Boezaart bleeding score were collected. Minimum absolute convergence and selection operator (LASSO) regression, as well as multivariate logistic regression, were used to determine the risk factors. A nomogram was developed in order to predict poor operating visibility during FESS, and its performance was evaluated utilizing both the training and verification datasets via various measures including receiver operating characteristic (ROC) curve analysis, area under the curve (AUC), Hosmer-Lemeshow goodness-of-fit test, calibration curve, and decision curve analysis.

**Results:**

Of the 369 patients who met the inclusion criteria, 88 of them exhibited POV during FESS. By deploying LASSO and multivariate logistic regression analyses, six risk factors were identified and used to construct a nomogram for predicting POV during FESS. These factors include prothrombin time (PT), prothrombin activity (PTA), Lund-Mackay score (LMS), Lund-Kennedy score (LKS), anesthetic method, and intraoperative hypertension. The AUC of the training set was found to be 0.820 while that of the verification set was 0.852. The Hosmer-Lemeshow goodness-of-fit test and calibration curve analysis revealed good consistency between predicted and actual probabilities. Also, the decision curve demonstrated that the nomogram had a high degree of clinical usefulness and net benefit.

**Conclusion:**

The constructed nomogram has a strong ability to predict the poor intraoperative field in patients with chronic rhinosinusitis, which can help preoperative judgment of high-risk patients and provide evidence for perioperative management and preoperative plan formulation.

## Background

Functional endoscopic sinus surgery (FESS) is a common technique for treating chronic rhinosinusitis (CRS) (1, 2). With improved therapeutic measures, bleeding during FESS has decreased (3), but some patients still experience significant bleeding during the surgery. The surgeon must frequently suction the blood to obtain a clear operative field, which not only prolongs the surgical time but also increases the incidence of complications and incomplete surgery (4, 5). Techniques such as intraoperative warm saline irrigation (6), local use of vasoconstrictors (such as epinephrine) (7), and controlled hypotension (8) can reduce the bleeding rate during FESS. However, these techniques are not sufficient to completely resolve unsatisfactory operative fields. Poor operative visibility (POV) during FESS remains a challenge for the surgeons.

Preoperative glucocorticoid use is an important measure for reducing unsatisfactory operative fields during FESS (9). Nevertheless, there is no unified standard for its dosage and timing (10). The objective of this investigation is to develop a predictive model for POV during FESS, detect CRS patients with poor operative fields, and provide evidence for preoperative management, which encompasses the administration of glucocorticoids.

## Data and methods

### Patients and study design

This retrospective analysis evaluates patients with chronic rhinosinusitis (CRS) (10) who underwent FESS from January 1, 2019, to December 31, 2022. Consistent with clinical guidelines, standardized pre- and perioperative management was provided to optimize outcomes and manage complications (11). Oral corticosteroids were mainly prescribed for patients with CRS and nasal polyps (CRSwNP), due to their efficacy in reducing polyp size and intraoperative bleeding, thus improving surgical outcomes. Nasal polyps were confirmed via endoscopic examination and CT scans (11).

Inclusion criteria and FESS indications (11): (1) Documented presence of significant anatomical abnormalities affecting the ostiomeatal complex or drainage of any paranasal sinus(es), (2) Evidence of nasal polyps affecting the ostiomeatal complex or drainage of the paranasal sinuses, (3) Patients had completed a minimum of 12 weeks of standardized medical treatment without satisfactory symptom improvement, and (4) Presence of complications such as intracranial or orbital involvement.

Exclusion criteria: (1) Severe cardiovascular system diseases, including heart failure or chronic hypotension, (2) Hematological or blood system diseases, (3) Coagulation disorders, (4) Use of anticoagulant drugs, including aspirin, (5) Abnormal liver and kidney function, (6) Nasal and sinus tumors or hyperthyroidism, (7) History of transfusion, (8) Malignant tumors, (9) Prior surgery on the nasal cavity or sinuses.

Surgical approach: Based on a comprehensive patient evaluation that included overall health, pain tolerance, and risks associated with head movement, we opted for either general or local anesthesia. For general anesthesia, we employed a combined intravenous-inhalation anesthesia (CIVIA) approach for FESS. This protocol involved the intraoperative use of sevoflurane and propofol, ensuring optimal patient comfort and safety without compromising surgical accuracy. In compliance with the established principles of FESS, our surgical team meticulously utilized a 1:10,000 dilution of epinephrine to achieve localized vasoconstriction, irrespective of the administration of general or local anesthesia. FESS underscores the importance of mucosal preservation, crucial for sustaining sinus function and mitigating postoperative complications. Throughout the procedure, surgeons utilized precision instruments to reduce trauma to the mucosa and surrounding healthy structures. The Anterior-to-Posterior Approach, following the Messerklinger Technique, ensured a methodical removal of pathological tissues, in strict accordance with ESS tenets.

**Figure 1:**
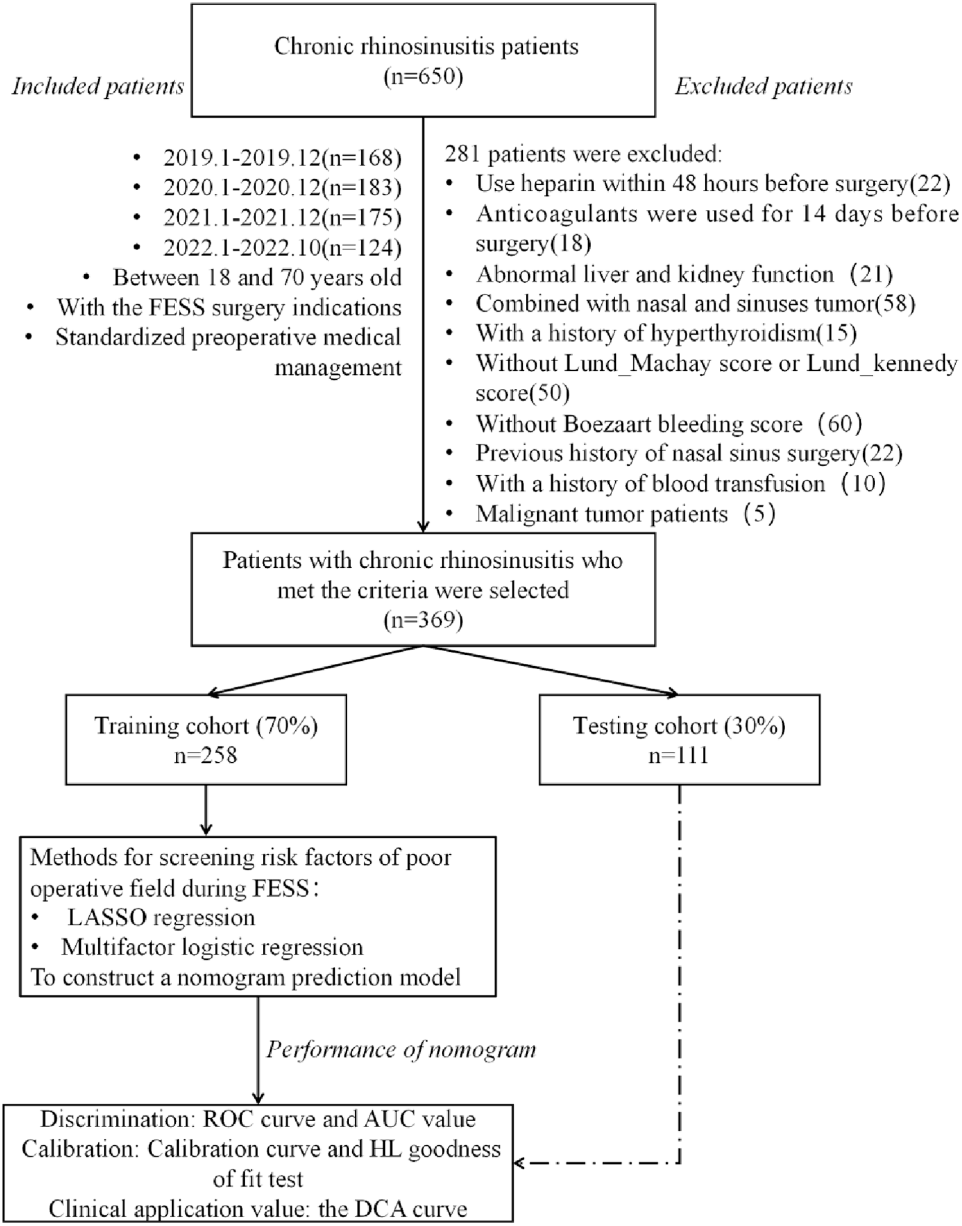
Flow chart of data collection and study design. FESS, functional endoscopic sinus surgery; ROC, receiver operating characteristic; AUC, area under curve; HL, Hosmer-Lemeshow; DCA, decision curve analysis.

The study aims to identify predictors of POV during FESS, develop a nomogram, and assess its performance in training and validation sets, as outlined in Figure 1.

### Collection and definition of Boezaart bleeding scores

This study involved meticulous collection and definition of Boezaart Bleeding Scores during FESS. The Boezaart score, ranging from 0 to 5, quantifies intraoperative bleeding, with higher scores indicating greater blood loss. Collection Process: Postoperatively, the primary surgeon records surgical findings, including the quality of the operative field and any complications. The Boezaart score, determined by the surgeon’s assessment of bleeding throughout the procedure, is recorded as part of the surgical record. Anesthesia Records: Concurrently, the anesthesia provider documents intraoperative details, such as the need for suctioning or vasoconstrictive agents, which supplement the Boezaart score by corroborating the surgeon’s evaluation of bleeding. Confirmation: A consensus meeting with the primary surgeon, anesthesia provider, and surgical assistant’s reviews and confirms the observed bleeding tendency, ensuring the Boezaart score accurately reflects the surgical experience. Documentation: The final Boezaart score is documented in the patient’s surgical record after this collaborative review, becoming part of their permanent medical record and used for postoperative analysis.

### Definition of POV

In accordance with existing literature (12) and our established data collection methodology, we have categorized a Boezaart score of 3 or higher as indicative of POV. This cutoff is selected to correspond to the clinical relevance of intraoperative bleeding; scores reaching this threshold and above suggest significant hemorrhage that can obstruct the surgeon’s view, thus requiring proactive intervention to ensure an unobstructed operative field.

Utilizing the Boezaart scoring system, which ranges from 0 to 5 points to assess the condition of the surgical field, a score of 3 or above is designated as POV (refer to Table 1 for detailed scoring criteria). This definition allows for a standardized approach to identifying and addressing situations where bleeding may compromise surgical outcomes.

**Table 1:** Boezaart score scale for bleeding in operating field.

Grade evaluation	Bleeding	Intraoperative area attraction frequency	Visibility of the surgical area	Field quality
0	None	–	–	Good
1	Slight	Hardly need attraction	–	
2	Slight	Need occasional attraction	–	
3	Slight	Need frequent attraction	Visible for a few seconds after attraction	Poor
4	Moderate	Need frequent attraction	Visible only immediately after attraction	
5	Serious	Need to constantly attract	The bleeding is faster than suction. The operation is almost impossible to perform.	

The following variables were included in the study: age(years), gender (female/male), residence type (rural/urban), marital status(married/unmarried or others), smoking (yes/no), drinking (yes/no), hypertension history (yes/no), use of nasal decongestants within 1 month before FESS (yes/no), concurrent allergic rhinitis status (yes/no), anesthesia method (local anesthesia[LA]/combined intravenous-inhalation anesthesia[CIVIA]). Intraoperative hypertension was defined as systolic pressure greater than 120 mmHg and/or diastolic pressure greater than 80 mmHg at the beginning of surgery (yes/no), while intraoperative heart rate was defined as normal (60–100 times/min), fast (>100 times/min), or slow (<60 times/min) at the beginning of surgery. Additionally, platelet count (PLT, 10^9/L), plasma prothrombin time (PT, sec), activated partial thromboplastin time (APTT, sec), thrombin time (TT, sec) and prothrombin activity (PTA, %) were recorded. The Lund-Mackay (LMS) and Lund-Kennedy (LKS) scores were applied for CT sinus scans and nasal endoscopies. The LMS (0–24 total) grades sinus opacification and ostiomeatal complex obstruction (0–12 per side), while the LKS (0–20 total) evaluates polyps, edema, discharge, crusting, and conchae hypertrophy (0–10 per side), guiding surgical management of sinonasal disease. The variables were listed in order of their hierarchy.

### Statistics

This study utilized R software (version 4.1.2) for statistical analysis. Normally distributed continuous variables were presented as mean (± standard deviation), and t-tests were used for intergroup comparisons. Non-normally distributed continuous variables were expressed as median (interquartile range), and the Mann–Whitney U test was used for intergroup comparisons. Categorical variables were presented as the number of cases (%), and intergroup comparisons were conducted using the chi-square test. Risk factors were screened using minimum absolute convergence and selection operator (LASSO) regression and multivariate logistic regression. Nomogram were drawn using the “rms” package. The performance and accuracy of nomogram were evaluated using receiver operating characteristic (ROC) curves, area under the curve (AUC), calibration curve evaluation, and the Hosmer-Lemeshow (HL) goodness-of-fit test. Decision curve analysis (DCA) was applied to assess the clinical usefulness and net benefit of prediction models. The threshold for statistical significance was set at *p* < 0.05.

## Result

### Basic characteristics of patients in training cohort and test cohort

A total of 369 CRS patients (218 males and 151 females, mean age 40.7 years) met the inclusion criteria, with 23.8% experiencing POV. In addition, 26.6% of patients had allergic rhinitis, 36.9% had utilized nasal decongestants within 1 month before FESS, and 25.2% presented with hypertension. The LMS and LKS were 14.0 ± 3.31 and 13.7 ± 2.63, respectively, with no statistically significant difference detected between the training set and validation set for all variables (*p* > 0.05) (refer to Table 2).

**Table 2:** Basic clinical features of CRS patients in training set and validation set.

	All	Training set	Validation set	t/*χ*^2^	*p*
	*N* = 369	*N* = 111	*N* = 258		
Intraoperative visual field				*χ^2^* = 0.292	0.589
Good	281 (76.2%)	82 (73.9%)	199 (77.1%)		
Bad	88 (23.8%)	29 (26.1%)	59 (22.9%)		
Age(years)	40.7 (15.7)	39.7 (15.7)	41.1 (15.7)	*χ^2^* = 0.826	0.412
Gender					0.657
Female	151 (40.9%)	43 (38.7%)	108 (41.9%)		
Male	218 (59.1%)	68 (61.3%)	150 (58.1%)	*χ^2^* = 0	
Marital status					0.966
Married	225 (61.0%)	67 (60.4%)	158 (61.2%)		
Unmarried or others	144 (39.0%)	44 (39.6%)	100 (38.8%)	*χ^2^* = 0.002	
Smoking					0.339
Yes	62 (16.8%)	15 (13.5%)	47 (18.2%)		
No	307 (83.2%)	96 (86.5%)	211 (81.8%)	*χ^2^* = 0.11	
Drinking					0.816
Yes	59 (16.0%)	19 (17.1%)	40 (15.5%)		
No	310 (84.0%)	92 (82.9%)	218 (84.5%)	*χ^2^* = 0	
Hypertension history					0.364
Yes	93 (25.2%)	24 (21.6%)	69 (26.7%)		
No	276 (74.8%)	87 (78.4%)	189 (73.3%)	*χ^2^* = 1.729	
Residence					1.000
Rural	199 (53.9%)	60 (54.1%)	139 (53.9%)		
Urban	170 (46.1%)	51 (45.9%)	119 (46.1%)		
Concurrent allergic rhinitis				*χ^2^* = 0	1.000
No	271 (73.4%)	82 (73.9%)	189 (73.3%)		
Yes	98 (26.6%)	29 (26.1%)	69 (26.7%)		
Use of nasal decongestants				*χ^2^* = 0.58	0.740
No	233 (63.1%)	72 (64.9%)	161 (62.4%)		
Yes	136 (36.9%)	39 (35.1%)	97 (37.6%)		
Anesthesia method				*χ^2^* = 0.915	1.000
Local	165 (44.7%)	50 (45.0%)	115 (44.6%)		
Aspiration compound	204 (55.3%)	61 (55.0%)	143 (55.4%)		
Intraoperative heart rate				*χ^2^* = 0.054	0.421
Normal	232 (62.9%)	65 (58.6%)	167 (64.7%)		
Fast	75 (20.3%)	27 (24.3%)	48 (18.6%)		
Slow	62 (16.8%)	19 (17.1%)	43 (16.7%)	*χ^2^* = 0.197	
Intraoperative hypertension					0.446
No	180 (48.8%)	58 (52.3%)	122 (47.3%)		
Yes	189 (51.2%)	53 (47.7%)	136 (52.7%)	*t* = −0.822	
PLT (10^*9^/L)	240 (62.8)	244 (67.1)	239 (61.0)	*t* = 0.704	0.482
PT (sec)	10.3 (1.12)	10.4 (1.14)	10.3 (1.12)	*t* = 0.851	0.396
PTA (%)	95.2 (9.48)	94.8 (9.80)	95.3 (9.35)	*t* = −0.455	0.650
TT (sec)	17.5 (0.96)	17.5 (0.86)	17.5 (1.01)	*t* = 0.0979	0.922
APTT (sec)	24.8 (3.52)	25.0 (3.38)	24.7 (3.57)	*t* = 0.729	0.467
LMS	14.0 (3.31)	14.0 (3.46)	14.0 (3.25)	*t* = 0.03	0.976
LKS	13.7 (2.63)	13.6 (2.55)	13.7 (2.66)	*t* = −0.224	0.823

PLT, platelet count; PT, plasma prothrombin time; APTT, activated partial thromboplastin time; TT, thrombin time; PTA, prothrombin activity.

### Risk factor screening

**Figure 2:**
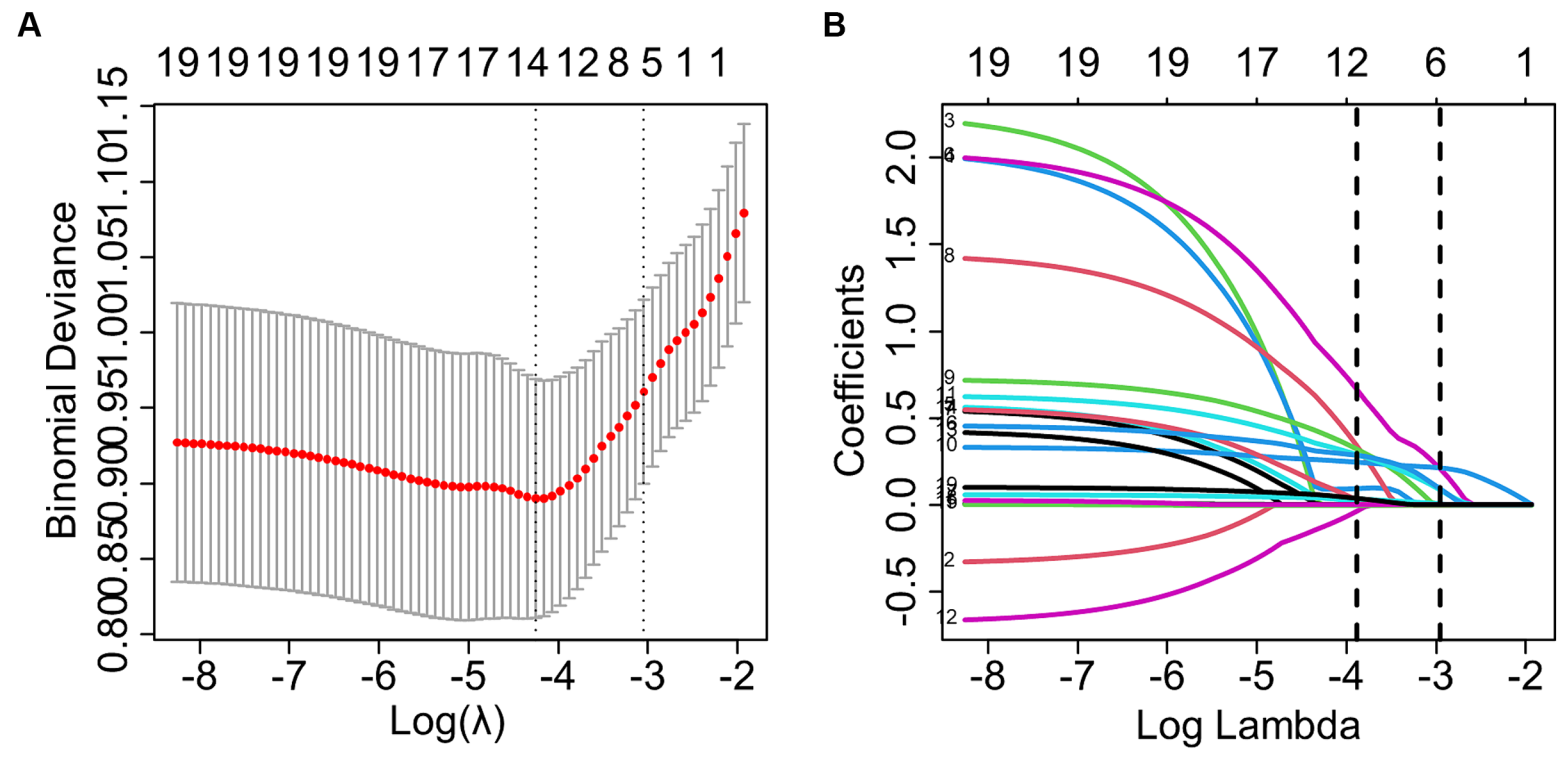
LASSO regression screening for predictors. **(A)** The graph depicting the relationship between the likelihood deviation (binomial deviation) and the log (λ) showed that the smallest value among all λ values was the minimum standard of 1se (1 standard error) at 6, which served as the criterion for selecting the optimal non-zero coefficient variable. **(B)** The LASSO coefficient curve for variables selection based on the minimum standard of 1se resulted in the identification of 6 non-zero coefficient variables.

LASSO regression was utilized to select the optimal λ and log (λ), resulting in a decrease in the number of predictive factors from 19 to 6. These 6 factors were identified as PT, PTA, LMS, LKS, anesthetic method, and intraoperative hypertension (please refer to Figure 2). Based on the findings presented in Table 3, the results of the multivariate logistic regression analysis revealed a significant association between POV during FESS and CIVIA, intraoperative hypertension, PT, PTA, LMS, and LKS (*p* < 0.05). Among these variables, odds ratios (OR) greater than 1 represented an increased risk factor, while ratios less than 1 were considered protective factors.

**Table 3:** Multivariate logistic regression analysis.

	*r*	Wald	OR(95%CI)	*p*
Anesthesia method				0.001
Local	*Ref*	*Ref*	*Ref*	
Inhalation compound	2.177	10.787	8.823 (2.548 ~ 34.735)	
Intraoperative Hypertension				0.013
No	*Ref*	*Ref*	*Ref*	
Yes	1.569	6.165	4.802 (1.439 ~ 17.478)	
PT	0.443	3.874	1.558 (1.009 ~ 2.453)	0.049
PTA	0.056	5.325	1.058 (1.009 ~ 1.111)	0.021
LMS	0.331	10.192	1.392 (1.141 ~ 1.718)	0.001
LKS	0.642	13.25	1.9 (1.369 ~ 2.745)	<0.001

PT, plasma prothrombin time; PTA, prothrombin activity.

**Figure 3:**
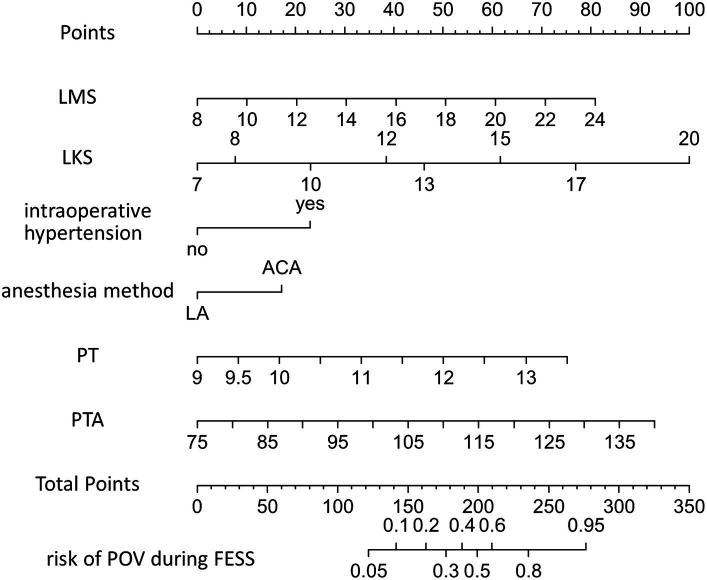
Nomogram used to predict the risk of POV during FESS. LMS, Lund-Machay score; LKS, Lund-Kennedy score; LA, local anesthesia; CIVIA, combined intravenous-inhalation anesthesia; PT, plasma prothrombin time; PTA, prothrombin activity; POV, poor operative visibility; FESS, functional endoscopic sinus surgery.

A nomogram for predicting FESS POV was constructed using multivariate logistic regression based on six variables selected through LASSO regression (see Figure 3). To utilize the nomogram, first identify the value for each of the six variables and draw a line upwards from that point on the respective variable axis. Next, sum the points obtained from all six variables to obtain the total points. Then, locate the total points on the “Total Points” axis and draw a perpendicular line downwards from the corresponding point to obtain the predicted risk probability of POV during FESS.

### Validation of the POV during FESS nomogram

**Figure 4:**
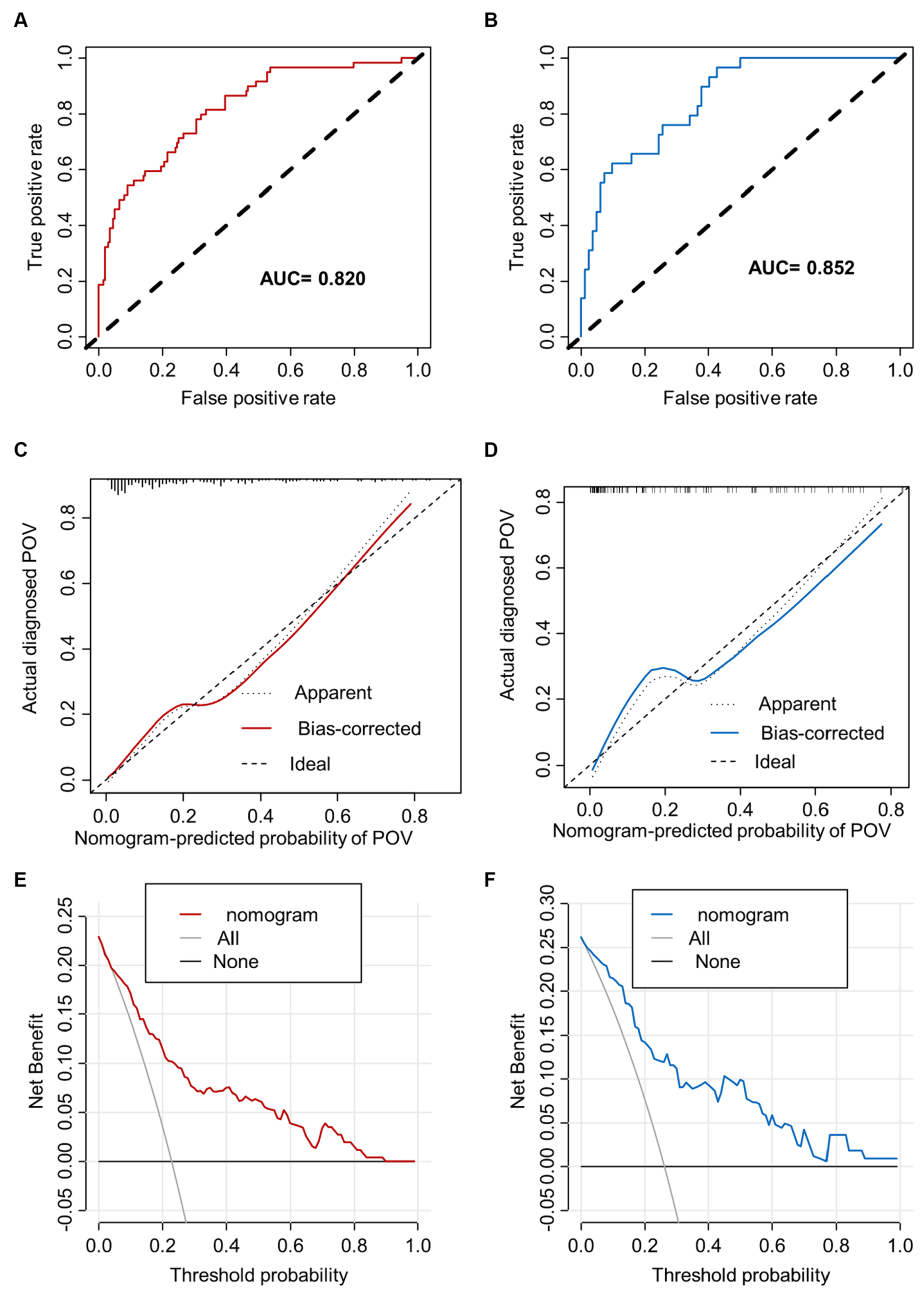
The performance of nomogram. ROC curves and AUC were utilized to evaluate the discriminative ability of the nomogram in both the training **(A)** and validation **(B)** cohorts. The calibration curve of the nomogram in the training **(C)** and validation **(D)** cohorts, respectively, solid line represents original curve, dotted line represents calibrated curve and diagonal line represents ideal curve, the closer to diagonal line indicates better prediction ability. The DCA, which uses **(E)** to represent the training dataset and **(F)** for the validation dataset, displays net benefit on the y-axis and threshold probability on the x-axis. It includes a dotted line that signifies all patients with CRS undergoing FESS who experience POV, and a thin black line that represents the hypothetical scenario of patients with CRS undergoing FESS without POV.

The training set nomogram achieved an AUC of 0.820 (95% confidence interval [CI], 0.760–0.880), while the validation set had an AUC of 0.852 (95% CI, 0.780–0.925), suggesting excellent discrimination ability of the model. The calibration graphs also showed good consistency between predicted and observed results in both the training and validation cohorts. Furthermore, the Hosmer-Lemeshow (HL) goodness-of-fit analysis produced a chi-square value of 7.6566 (*p* = 0.4677) in the training cohort and a chi-square value of 7.4562 (*p* = 0.4883) in the validation cohort, further confirming the calibration ability of the model. Additionally, as shown in Figure 4, the prediction nomogram model demonstrated considerable net benefits across most threshold probabilities at various time points, indicating its potential clinical application value. Therefore, the nomogram performed well in both the training and validation cohorts.

## Discussion

Maintaining a clear surgical field is crucial for the success of FESS, as even slight intraoperative bleeding can significantly affect the procedure (13). To help predict the likelihood of an unsatisfactory surgical field, we developed a nomogram using six variables selected through lasso and multivariate logistic regression methods. Our validation results showed that the nomogram had excellent discriminative and calibration abilities. Risk factors for an unsatisfactory surgical field included CIVIA and intraoperative hypertension, while the LMS, LKS, PT, and PTA were positively correlated with the risk.

General anesthesia is commonly used in FESS due to its advantages of patient stillness, airway management, sedation, and control over blood pressure and heart rate (14). There are different methods of administering general anesthesia such as total intravenous anesthesia (TIVA) or CIVIA with sevoflurane. Previous studies have shown that total intravenous propofol anesthesia can reduce bleeding during FESS and improve the surgical field (15–17). However, our study suggests that the use of CIVIA with sevoflurane may increase the risk of an unsatisfactory surgical field. Consistent with previous research (18), our findings suggest that avoiding inhalation anesthesia (sevoflurane) can improve the quality of the surgical field during FESS.

Controlling hypotension has been recognized as an effective strategy to reduce bleeding during FESS (8, 19). Our decision to incorporate “intraoperative hypertension” as a variable, rather than preoperative blood pressure, was driven by its closer reflection of the cardiovascular status and associated bleeding risks encountered during functional endoscopic sinus surgery (FESS). The blood pressure reading immediately prior to the initial surgical incision is especially significant, offering critical information to the surgeon for operative field management. The findings from this study indicate that an increase in intraoperative blood pressure, regardless of the patient’s hypertensive history, may compromise the quality of the surgical field. Under local anesthesia, the experience of negative emotions such as anxiety and tension can trigger sympathetic nerve activation and increase systolic blood pressure and heart rate (20). For patients receiving local anesthesia, it is recommended to conduct psychological assessments and provide education on stress reduction techniques. In addition, beta-blockers may be applied to reduce cardiac output and lower blood pressure, which can ultimately improve the quality of the surgical field (21), as per the formula “mean arterial pressure (MAP) = systemic vascular resistance (SVR) * cardiac output (CO) + central venous pressure (CVP)” (22). In summary, our study, combined with previous researches, supports the notion that intraoperative hypertension significantly increases the risk of POV during FESS.

The LMS (23) is widely used as an objective measure to assess sinus and nasal inflammation in patients. It has been shown to be closely associated with the severity of clinical symptoms and is also considered as a valuable prognostic indicator for FESS (23, 24). Bai et al. (25) found a significant positive correlation between the LMS and bleeding during FESS. Moreover, the LKS is another reliable tool for assessing CRS, wherein higher scores indicate more severe CRS inflammation (26). This study highlights that the severity of CRS inflammation, as indicated by the higher LMS and LKS, is a risk factor for POV during FESS.

Coagulation function tests have been found to be useful indicators for the prediction of thrombus formation and heat stroke prognosis (27, 28). However, a study involving 88 adult CRS patients who underwent FESS revealed that preoperative coagulation screening tests such as PT, international normalized ratio (INR), and APTT were not effective in predicting intraoperative bleeding (29). Nevertheless, other studies have indicated that the use of anticoagulants, either locally or systemically, can help to reduce bleeding associated with FESS (30–32). Coincidentally, our investigation has revealed that even when PT and PTA were within the normal range, their elevation was significantly associated with POV during FESS. Therefore, our research highlights the necessity for preoperative coagulation function tests, particularly PT and PTA, as predictive factors for bleeding during FESS.

In this study, we have demonstrated the potential clinical utility of a nomogram based on easily obtainable predictors, which, once validated, could significantly enhance preoperative assessment for FESS. Despite the promise shown by our predictive tool, several limitations must be acknowledged to guide future research.

The study’s scope was confined to patients undergoing CIVIA, precluding the assessment of other general anesthesia techniques that could influence bleeding risks and surgical outcomes. This restriction may affect the nomogram’s broader applicability across different anesthesia practices. While our endoscopic scoring system included an evaluation of nasal polyps, and glucocorticoid treatment was employed in preoperative management to reduce polyp size, our study did not specifically analyze the impact of these treatments on bleeding in patients with nasal polyps. Furthermore, our research did not account for the various subtypes of chronic rhinosinusitis (CRS), including CRS with nasal polyps (CRSwNP) and CRS without nasal polyps (CRSsNP), nor did it consider eosinophil-predominant type 2 inflammatory subtypes and aspirin-exacerbated respiratory disease (AERD). This represents a significant gap, as these conditions and the specific effects of glucocorticoids could markedly influence intraoperative bleeding patterns and surgical management. Addressing these factors in future studies is crucial to refine our predictive models and align them more closely with clinical practice.

Additionally, during the data analysis, we excluded 40 patients who were on anticoagulant medications, a decision made to minimize confounding effects on surgical procedures and bleeding risks. Although this group was small, we recognize that their exclusion may have introduced selection bias, potentially skewing our findings if patients at lower predicted bleeding risk were more often maintained on these medications. Expanding future research to include these patients is crucial for a comprehensive evaluation of perioperative bleeding risks and for informing balanced clinical decisions regarding anticoagulation management.

The study’s design, characterized by its single-center and retrospective approach, primarily involved Chinese adult patients, which may constrain the generalizability of our findings. We acknowledge that incomplete LMS and LKS data for certain patients, a consequence of inconsistent documentation practices and the inherent limitations of a retrospective review, could potentially bias our analysis. These scores are essential for evaluating the severity of sinus disease and inflammation, critical factors influencing intraoperative bleeding and visibility. To address these limitations, we have diligently sought to ensure the robustness of our analysis, grounding our conclusions in the available data. Looking ahead, we are committed to standardizing data collection protocols in future studies to ensure a more comprehensive and diverse dataset. This will be instrumental in facilitating multi-center clinical trials, thereby enhancing the nomogram’s external validity and broadening its clinical applicability.

In refining our understanding of bleeding tendencies, it is imperative to consider both expected and unexpected parameters. Expected parameters might include known factors such as the severity of inflammation, as indicated by the LMS and LKS, which have been correlated with increased bleeding during FESS. Unexpected parameters could involve individual patient factors, such as genetic predispositions or unanticipated comorbidities, which were not accounted for in our current model. A more granular analysis of these factors will be crucial in future studies to provide a more accurate prediction of bleeding risk.

## Conclusion

In conclusion, this study successfully developed and evaluated a nomogram for predicting POV during FESS. Six risk factors were identified using LASSO and multivariate logistic regression, and the nomogram exhibited good performance with ROC curve analysis, AUC, Hosmer-Lemeshow goodness-of-fit test, calibration curve, and DCA. It is expected that this nomogram will be useful in clinical practice for predicting poor FESS operative visibility and improving patient outcomes.

## Data availability statement

The original contributions presented in the study are included in the article/supplementary material, further inquiries can be directed to the corresponding author.

## Ethics statement

The studies involving humans were approved by the Ethics Committee of the Fourth Clinical College of Chongqing Medical University the protocol with the approval number was LL-202231. The studies were conducted in accordance with the local legislation and institutional requirements. Written informed consent for participation was not required from the participants or the participants’ legal guardians/next of kin in accordance with the national legislation and institutional requirements.

## Author contributions

DS: Conceptualization, Data curation, Formal analysis, Investigation, Methodology, Project administration, Software, Supervision, Visualization, Writing – original draft, Writing – review & editing. YL: Data curation, Formal analysis, Investigation, Methodology, Software, Writing – original draft, Writing – review & editing. FY: Conceptualization, Data curation, Formal analysis, Software, Supervision, Visualization, Writing – original draft, Writing – review & editing. LL: Conceptualization, Data curation, Methodology, Software, Validation, Writing – original draft, Writing – review & editing. XM: Conceptualization, Data curation, Methodology, Software, Writing – original draft, Writing – review & editing. XX: Conceptualization, Data curation, Software, Writing – original draft, Writing – review & editing.

## Glossary

FESS: functional endoscopic sinus surgery
CRS: chronic rhinosinusitis
POV: poor operative visibility
LMS: Lund-Machay score
LKS: Lund-kennedy score
CIVIA: combined intravenous-inhalation anesthesia
LASSO: minimum absolute convergence and selection operator
ROC: receiver operating characteristic
AUC: area under the curve
DCA: decision curve analysis
HL: Hosmer-Lemeshow
PLT: platelet count
PT: plasma prothrombin time
APTT: activated partial thromboplastin time
TT: thrombin time
PTA: prothrombin activity
OR: odds ratio
CI: confidence interval
TIVA: total intravenous anesthesia
MAP: mean arterial pressure
SVR: systemic vascular resistance
CO: cardiac output
CVP: central venous pressure
INR: international normalized ratio
AERD: aspirin-exacerbated respiratory disease
